# Sex Differences in Sarcopenia in Patients Undergoing Total Knee Arthroplasty for Advanced Knee Osteoarthritis

**DOI:** 10.3390/medicina60020226

**Published:** 2024-01-28

**Authors:** Oog-Jin Shon, Gi Beom Kim, Seong Hyeon Jo

**Affiliations:** 1 Department of Orthopedic Surgery, College of Medicine, Yeungnam University, 170 Hyonchung-ro, Namgu, Daegu 42415, Republic of Korea; maestro-jin@hanmail.net; 2 Department of Orthopedic Surgery, Yeungnam University Medical Center, 170 Hyonchung-ro, Namgu, Daegu 42415, Republic of Korea; fmsndkfmoo@gmail.com

**Keywords:** total knee arthroplasty, sarcopenia, patient-reported outcome measures, sex differences

## Abstract

*Background and Objectives:* The purpose of this study was to compare sex differences in the incidence of sarcopenia, demographic characteristics, and preoperative sarcopenic parameters in patients undergoing TKA for advanced knee osteoarthritis (OA). Moreover, we sought to compare patient-reported outcome measures (PROMs) and the predisposing factors after TKA in patients with sarcopenia by sex through subgroup analysis. *Materials and Methods:* From May 2020 to September 2022, a total of 892 patients who were evaluable for sarcopenia before primary TKA were enrolled. Sarcopenia was defined according to the Asian Working Group for Sarcopenia 2019 criteria. Patients were assessed according to the presence or absence of sarcopenia. After a two-to-one matched-pair analysis for subgroup analysis, 21 knees in men were matched with a corresponding number of knees in women (42), resulting in a total of 63 knees. PROMs were investigated using the Knee Injury and Osteoarthritis Outcome Score, Western Ontario and McMaster Universities Osteoarthritis Index, and the Short Form-12 physical and mental component summary scores. Moreover, the postoperative complications and predisposing factors for male sarcopenia were investigated. *Results:* The prevalence of sarcopenia was 10.9% (97/892), and the prevalence was higher in men (19.6%, 21/107) than in women (9.7%, 76/785). In subgroup analyses, male patients had significantly inferior PROMs up to 12 months after index surgery. Moreover, there was no significant difference in the systemic complications between the two groups. Multivariate binary logistic regression analysis indicated that alcohol consumption, smoking, and higher modified Charlson Comorbidity Index (mCCI) were predisposing factors for male patients with sarcopenia. The prevalence of sarcopenia was higher in male patients undergoing primary TKA. *Conclusions:* When compared with the propensity-matched female group, male patients had inferior PROMs up to 12 months postoperatively. Alcohol consumption, current smoker status, and higher mCCI were predisposing factors for sarcopenia in male patients with advanced knee OA.

## 1. Introduction

Skeletal muscle naturally declines with age, and this loss accelerates after the age of 65, which can increase the risk of poor quality of life, physical disability, and death [1]. Sarcopenia, defined as the loss of muscle mass, strength, and function with aging [2] is known to be an independent risk factor for frailty, falls, and lower extremity fractures [1,3,4]. In 2020, the Asian Working Group for Sarcopenia (AWGS) published updated guidelines based on East Asian and Southeast Asian studies (AWGS 2019) [5]. As sarcopenia has been associated with a variety of total knee arthroplasty (TKA)-related complications including periprosthetic joint infection, its importance should be further emphasized in elderly patients undergoing TKA [6,7,8].

Elderly women account for about 70–90% of TKA candidates, and their number is gradually increasing due to the aging population. As some studies have reported, especially in Asia, this female predominance is more pronounced [9]. Therefore, to date, most studies related to sarcopenia have focused on elderly women, and their outcomes have mostly addressed these women [10]. However, with the gradual increase in TKA volume [11] and since the number of elderly men who are undergoing TKA is also increasing it has become necessary to pay attention to the results of TKA in the elderly men group. However, few studies have been conducted on the comparison of outcomes after TKA in elderly men and women with sarcopenia and on the risk factors in elderly men with sarcopenia.

The purpose of this study was to compare sex differences in the incidence of sarcopenia, demographic characteristics, and preoperative sarcopenic parameters in patients undergoing TKA for advanced knee OA. Moreover, we also sought to compare PROMs and the predisposing factors after TKA in patients with sarcopenia by sex through subgroup analysis. We hypothesized that (1) the incidence, preoperative parameters, and predisposing factors for sarcopenia in patients undergoing TKA for advanced knee OA would significantly differ between the sexes; (2) when compared with the propensity-matched female control group, inferior improvement would be reported for the male study group.

## 2. Materials and Methods

### 2.1. Study Subjects

**Figure 1:**
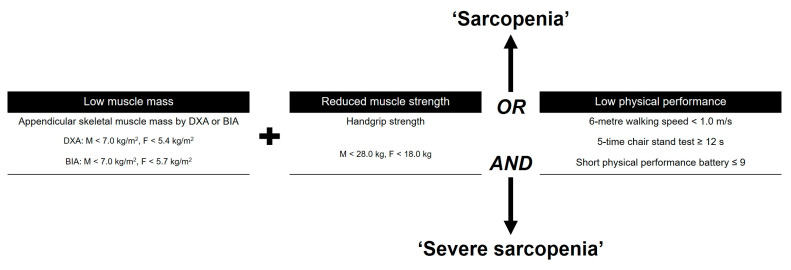
Guideline for the Asian Working Group for Sarcopenia (AWGS) 2020. DXA, dual-energy X-ray absorptiometry; BIA, bioelectrical impedance analysis.

After obtaining the approval of the institutional review board of our hospital, we conducted a retrospective comparative, single-center study. From May 2020 to September 2022, a consecutive series of 922 patients (1270 knees) who underwent primary TKA were screened. Patients were enrolled who were 60 years of age or older, had symptomatic progressive osteoarthritis (OA) (Kellgren–Lawrence (K–L) grade ≥ 3), had a follow-up period of at a minimum of 1 year after index surgery, and were able to be evaluated using preoperative assessments for sarcopenia, including body composition, muscle strength, and physical performance, based on AWGS 2019 criteria (Figure 1) [5].

Patients with other diagnoses, such as rheumatoid arthritis or post-traumatic OA, patients who had not been able to walk independently within the previous year because of medical comorbidities (functionally dependent) [12], whose body composition could not be measured because of body metal implants in the appendicular region of the body, and who were severely obese (body mass index (BMI) ≥ 35 kg/m^2^) were excluded.

### 2.2. Definition of Sarcopenia

Subjects who had low muscle mass, low muscle strength, and/or low physical performance were classified as having sarcopenia. The skeletal muscle index was measured using whole-body dual-energy X-ray absorptiometry (DXA) (Horizon, Hologic, Bedford, MA, USA) [13]. Appendicular skeletal muscle mass (ASM) was calculated as the sum of arm and leg lean muscle mass [13]. As suggested by Baumgartner et al. [14], ASMI was derived from DXA measurements by dividing ASM by height squared (kg/m^2^). Low muscle mass was categorized as ASMI < 5.4 kg/m^2^ for women and <7.0 kg/m^2^ for men [5]. Isometric handgrip strength was assessed with a grip strength test using a dynamometer (Jamar, Bolingbrook, IL, USA) [15,16]. Low strength was categorized as a handgrip strength < 28 kg for men and <18 kg for women [5]. Physical performance was measured with a walking speed of 6 m; a walking speed of <1.0 m/s for both men and women was defined as low physical performance [5].

### 2.3. Matched-Pair Analysis

**Figure 2:**
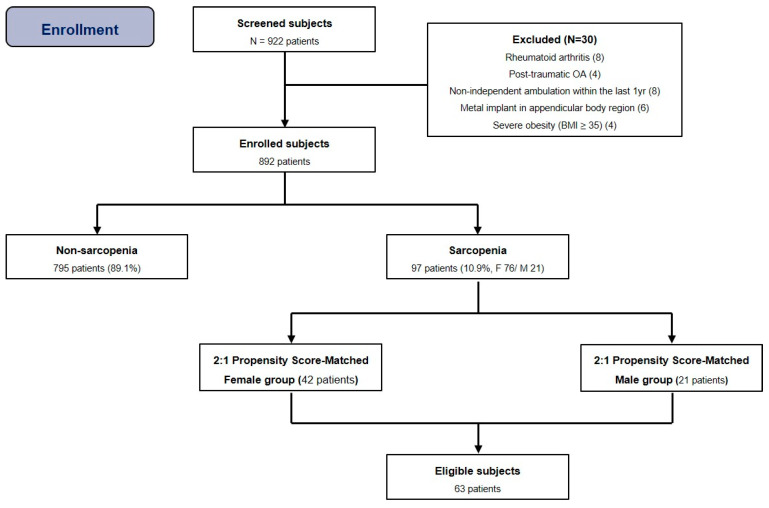
Flow diagram illustrating patient enrollment. Ultimately, 892 patients were enrolled in our study. After 2:1 propensity score matching, 63 patients were analyzed. Note: BMI, body mass index.

Since attempting to match two untreated subjects to each treated subject would improve precision without a commensurate increase in bias or confounding factors in some settings, we used the two-to-one matched-pair analysis. Based on previous relevant studies [10,17], propensity scores were estimated from multiple logistic regression analyses including all relevant covariates. The matching criteria were age at surgery and BMI. Two-to-one matched-pair analysis was performed using nearest neighbor matching, a propensity score (PS) matching method [18]. All subjects were grouped as either women (F) or men (M), and we conducted matching in the order of the smallest absolute value of the difference in the propensity score (Figure 2).

When the standardized difference was <0.1 in determining the balance of covariance, it was regarded as balanced matching.

### 2.4. Operative Details and Postoperative Protocol

All operations were conducted by two experienced orthopedic surgeons in our hospital using the modified gap-balancing technique with the same implant (posterior-stabilized, Attune^®^ TKA System, Depuy Synthes Inc., Warsaw, IN, USA) [19]. All prostheses were used with cement, and fixed-bearing antioxidant polyethylene inserts were used.

All patients received the same rehabilitation protocol. A closed suction drain was inserted and removed 24 h after the index surgery. The same perioperative pain control protocol was used, including a multimodal drug regimen, intraoperative periarticular injection, and postoperative patient-controlled analgesic device. Postoperative range of motion (ROM) was allowed on the day of surgery. After resolution of acute postoperative pain, partial weight-bearing with a crutch was allowed on the first postoperative day. Full weight-bearing was allowed 2 or 3 weeks after index surgery [20].

### 2.5. Outcome Assessment

All data were obtained from the institutional electronic medical records. Patient demographic characteristics (including age, sex, BMI, follow-up period, current smoking status, alcohol consumption, and modified Charlson Comorbidity Index (mCCI) [21]); and laboratory data (including hemoglobin (Hb, g/dL) and total protein (TP, g/dL) levels) were compared between the female (F) and male (M) groups. The mCCI was calculated as the sum of the weighted scores for each comorbidity [21] (Table 1).

Patients were regularly assessed preoperatively, and at 6 weeks, 6 months, and 12 months postoperatively, and annually thereafter. All clinical outcomes were compared between the groups (group F vs. M).

For assessments of PROMs, the Knee Injury and Osteoarthritis Outcome Score (KOOS) [22], the Western Ontario and McMaster Universities Osteoarthritis Index (WOMAC) [23], and the Short Form (SF)-12 physical and mental component summary scores [24] were investigated. The ROM of the knee joint was assessed using a standardized manual goniometer with a 30 cm long plastic movable arm (from the greater trochanter of the femur to the lateral malleolus) [25]. They were recorded by an independent researcher in the outpatient clinic.

Moreover, the incidence of systemic and specific complications was compared between the groups. Systemic complications were considered such as the worsening of an underlying systemic comorbidity or the development of a new medical problem [26]. Specific complications included the need for postoperative blood transfusion, venous thromboembolism (VTE), periprosthetic joint infection (PJI) [27], and periprosthetic fracture. Patients whose Hb level dropped to less than 7.0 g/dL within 2 weeks of the index surgery received a postoperative blood transfusion [28].

### 2.6. Statistical Analysis

A statistical evaluation was performed using SPSS version 28 software (IBM Corp, Armonk, NY, USA), and continuous data are expressed as means with range or ± standard deviation. The Kolmogorov–Smirnov test was used to evaluate all dependent variables for normality of distribution and equality of variance. Pearson’s two-tailed χ^2^ test or Fisher’s exact test was used for comparison of proportions between groups. Independent samples *t*-test was performed to detect significant differences between groups. Univariate and multivariate logistic regression analyses were used on categorical and continuous variables to assess for factors impacting the presence of sarcopenia in male patients with end-stage knee OA. For all tests, a *p* value < 0.05 was considered statistically significant.

## 3. Results

In total, this study included 892 patients (795 women, 97 men). The average age at surgery was 71.6 years (range, 60–88 years), and the average follow-up period was 24.5 months (range, 12.0–37.0 months) (Table 2).

The overall prevalence of sarcopenia in this cohort was 10.9% (97/892), and it was more common in male patients (21/107, 19.6%) than in female patients (76/785, 9.7%). After two-to-one matched-pair analysis for subgroup, 21 knees in men were matched with a corresponding number of knees in women (42), resulting in a total of 63 knees (Table 3).

**Figure 3:**
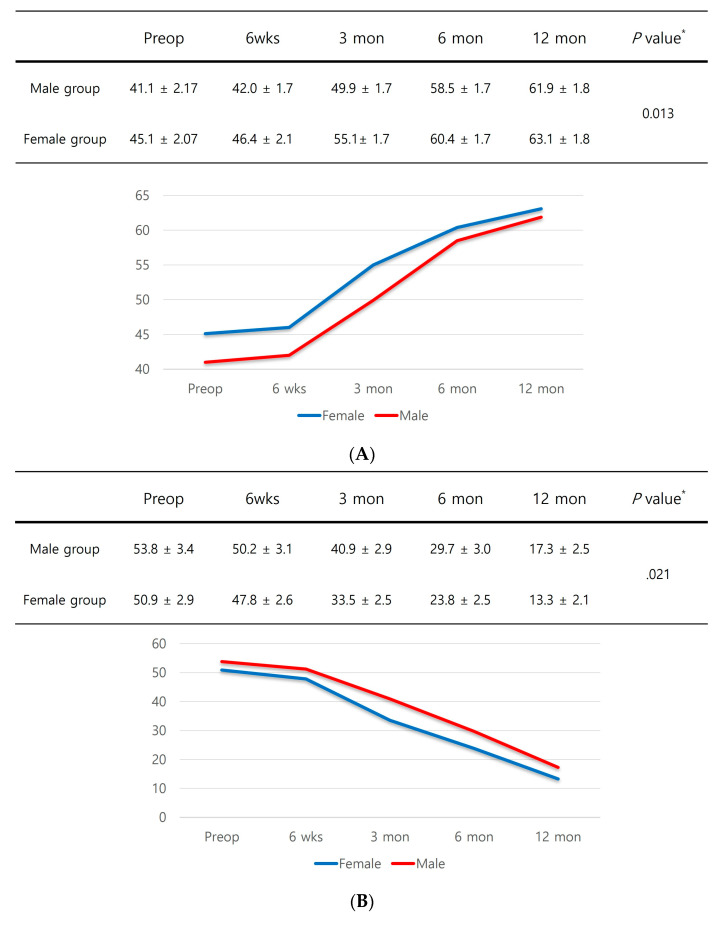

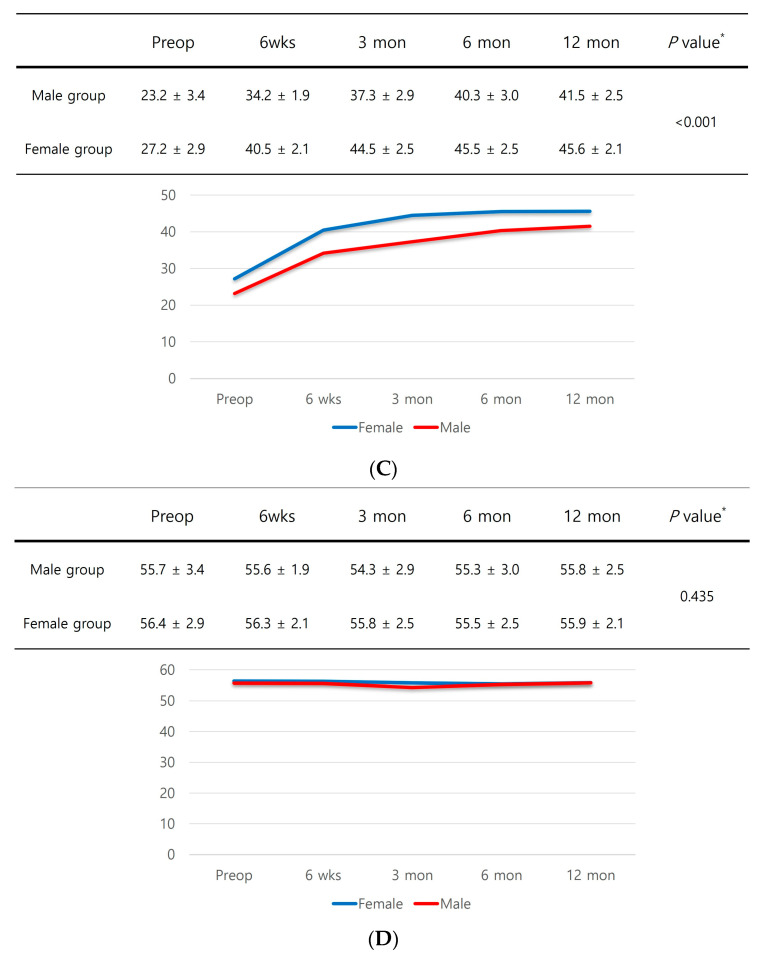
(**A**) KOOS score. (**B**) WOMAC score. (**C**) SF-12 physical component summary scores. (**D**) SF-12 mental component summary scores. * Independent samples *t*-test was performed to detect significant differences between groups.

In subgroup analyses, male patients with sarcopenia had significantly inferior PROMs up to one year after surgery compared to female patients with sarcopenia (Figure 3A–D).

Meanwhile, there was no significant difference in systemic complications between the two groups (Table 4).

The multivariate logistic regression analysis revealed that alcohol consumption and higher mCCI (OR, 1.4; 95% CI, 0.9–1.8; *p* = 0.003), current smoker status (OR, 1.2; 95% CI, 0.9–1.4; *p* = 0.019), and higher mCCI (OR: 1.2; 95% CI: 0.8–1.5; *p* = 0.036) were found to be predisposing risk factors for sarcopenia in male patients (Table 5).

## 4. Discussion

The most notable finding of the current study was that a propensity score-matched analysis showed inferior PROMs in male patients with sarcopenia undergoing TKA for advanced knee OA up to one year after index surgery. Furthermore, multivariate logistic regression analysis identified alcohol consumption, smoking, and higher mCCI as predisposing risk factors for sarcopenia in male patients in this cohort. To the best of our knowledge, some recent studies have reported the incidence or outcomes of sarcopenia in patients with knee OA [7,17,29]. However, few studies have reported sex differences in patients with sarcopenia undergoing TKA for advanced knee OA. Our study illustrates a higher prevalence of sarcopenia in male patients (19.6%) with end-stage OA of the knee joint compared to female patients (9.7%). This is consistent with the prevalence reported in studies of sarcopenia patients with musculoskeletal disorders. Studies that assessed the effectiveness of TKA in patients with end-stage knee OA with or without sarcopenia showed a high prevalence of sarcopenia in male patients (approximately 41.7%) compared to female patients (30.4%) [7]. Although in different disease entities, studies of sarcopenia in hip and distal radius fracture patients reported from Asia have all reported a higher prevalence in male patients than in female patients [30,31,32]. Another study analyzing factors associated with sarcopenia reported that male sex and smoking were independently associated with pre-sarcopenia, sarcopenia, and severe sarcopenia [33]. This study reported a higher prevalence of sarcopenia in older males than in females and suggested that the sex differences in prevalence may be due to sex differences in insulin growth factor-1 levels [33,34].

Notably, male patients with sarcopenia showed significantly inferior PROMs up to 1 year after TKA in the present study. Men typically gain an average of 40% more lean body mass and 60% more strength than women over the course of several decades of their lives [35]. As men typically maintain higher levels of physical activity and therefore higher levels of muscle strength than women [36], they may be more susceptible to muscle loss if their activity is reduced by knee OA. Female patients may be less susceptible to the adverse effects of limited physical activity due to knee OA because they are naturally less physically active than male patients. Therefore, for male sarcopenic patients with advanced knee OA, increasing muscle mass through regular exercise, especially resistance or strength training, along with eating a balanced diet that includes adequate proteins are crucial measures to prevent or slow down the progression of sarcopenia.

In the present study, a higher mCCI was found to be one of the predisposing factors for male sarcopenia patients with advanced knee OA. Several studies have reported that the mCCI reflects the overall health status and even the mortality rate based on the multiple comorbidities of a patient [21,37,38]. On average, male sarcopenia patients present poorer overall health status, as measured using the mCCI, which may compromise their ability to recover from muscle loss and physical activity limitations, compared to female patients with similar levels of knee OA. A study comparing mortality rates between the sexes for low-energy proximal femur fractures showed that men had a higher risk of death than women [37]. The study reported that men had a higher mean age-adjusted CCI and that this poorer health status could affect not only the fracture itself but also the ability to recover from the injury. A comparison of our with the multiple comorbidities found to be significantly more common in male sarcopenia with advanced knee OA suggests that a decreased overall health status may predispose these men to poorer functional recovery after TKA.

Lifestyle behaviors such as alcohol consumption and smoking have been identified as risk factors for muscle weakness due to loss of muscle mass and strength [39,40]. Although there is some variation based on nationality and culture, men are much more likely to drink alcohol than women. According to a study based on the 2010–2012 National China Nutrition and Health Survey, the prevalence of alcohol consumption was about four times higher among men than women [41]. Similarly, about 49.8% of adult men and 4.2% of adult women in South Korea were identified as smokers, according to the 2017 World Health Organization report [42]. Our results suggest that alcohol consumption and smoking may have detrimental effects on muscle mass in male patients undergoing TKA for advanced knee OA.

Despite these informative outcomes, there are some limitations associated with our study. First, this study had a relatively small sample size. This may be due to the fact that women more commonly undergo TKA for advanced knee OA in Asia than men [43], so less screening is conducted for sarcopenia in this limited number of male patients. Further studies in larger cohorts or community-dwelling populations are needed to confirm the findings of this study. Second, this study had a short follow-up period, which may have missed significant differences. In addition, the current results are not necessarily indicative of longer-term outcomes. Third, this study was not able to identify underlying causes, such as hormonal changes or reduced physical activity, for the sex differences in sarcopenia. As people age, many factors contribute to the development of sarcopenia, including hormonal changes, reduced physical activity, poor nutrition, and certain health conditions. However, this study showed that alcohol consumption, smoking, and mCCI may be predisposing factors in male sarcopenia patients with advanced knee OA. Further studies are needed to determine the relevance of hormonal or specific physical activity changes in men in relation to the progression of sarcopenia. Fourth, since this study only included patients with limited grades of OA (K-L grade III or IV), the results may not be comparable to those of all male patients with other grades of OA.

## 5. Conclusions

The prevalence of sarcopenia was higher in male patients undergoing primary TKA. When compared with the propensity-matched female group, male patients had inferior PROMs up to 12 months postoperatively. Alcohol consumption, current smoker status, and higher mCCI were predisposing factors for sarcopenia in male patients with advanced knee OA.

## Figures and Tables

**Table 1:** Scoring system for the modified Charlson Comorbidity Index (mCCI) [22].

Variables	Score
Peripheral vascular disease or pain at rest	1
Congestive heart failure	1
Prior myocardial infarction	1
Diabetes mellitus	1
Prior transient ischemic attack or stroke	1
Chronic obstructive pulmonary disease	1
Renal failure	2
Hemiplegia or paraplegia	2
Ascites or esophageal varices	3
Disseminated cancer	6
(Age)	
≤40 years old	0
41–50 years old	1
51–60 years old	2
61–70 years old	3
≥70 years old	4

Note: Different scores were assigned to certain comorbidities and age groups. The final score of the mCCI is the sum of the weighted scores for each comorbidity.

**Table 2:** Demographic characteristics according to the presence of sarcopenia.

	Total	Sarcopenia	Non-Sarcopenia	*p* Value
Patients, *n* (%) ^†^	892 (100)	97 (10.9)	795 (89.1)	-
Age (years) *	71.6 (60–88)	75.8 (69–88)	68.7 (60–76)	0.871
Sex, n ^†^				
Female, *n*	785 (88.0)	76 (78.4)	709 (89.2)	0.002
Male, *n*	107 (11.2)	21 (21.6)	86 (10.8)	
BMI (kg/m^2^) ^‡^	26.9 ± 3.7	23.2 ± 3.4	27.5 ± 3.2	<0.001
Mean f/u (mon) *	24.5 (12–37)	24.7 (12–37)	24.4 (12–37)	0.791
Bilaterality, *n* (%) ^†^	381 (42.7)	40 (41.2)	337 (42.4)	0.810
Preop K–L grade, *n* (%) ^†^				
Grade III	226 (25.3)	26 (26.8)	200 (25.2)	0.745
Grade IV	666 (74.7)	71 (73.2)	595 (74.8)	
Current smoker, *n* (%) ^†^	76 (8.5)	12 (12.4)	64 (8.1)	0.165
Alcohol drinker, *n* (%) ^†^	87 (9.8)	15 (15.5)	72 (9.1)	0.060
mCCI ^†^				
2	40 (4.5)	-	40 (5.0)	<0.001
3 and 4	419 (47.0)	7 (7.2)	412 (51.8)	
5–8	316 (35.4)	46 (47.4)	270 (34.0)	
≥9	117 (13.1)	44 (45.4)	73 (9.2)	
Hb level (g/dL) ^‡^	12.5 ± 2.1	11.1 ± 3.2	12.8 ± 1.0	0.021
Total protein (g/dL) ^‡^	6.8 ± 1.0	5.9 ± 1.2	7.0 ± 0.9	0.037
ASM index (ASM/height^2^), kg/m^2 ‡^	6.0 ± 0.6	5.0 ± 0.8	6.3 ± 0.6	0.004
Grip strength, kg ^‡^	18.0 ± 3.4	16.1 ± 4.5	19.2 ± 3.1	0.030
6 m walking speed, m/s ^‡^	1.2 ± 0.2	0.8 ± 0.1	1.4 ± 0.2	0.025

Note: BMI, body mass index; f/u, follow-up; Preop, preoperative; K-L, Kellgren–Lawrence; mCCI, modified Charlson Comorbidity Index; Hb, hemoglobin; ASMI, appendicular skeletal muscle mass index. * Values are provided as numbers with ranges. ^†^ Values are provided as numbers with percentages. ^‡^ Values are provided as means ± standard deviations.

**Table 3:** Sex differences of demographic characteristics in propensity-matched population.

	2:1 Propensity-Matched Population (*n* = 63)		
	Female Sarcopenia (*n* = 42)	Male Sarcopenia (*n* = 21)	*p* Value
Age (years) *	75.6 (70–83)	76.1 (70–82)	0.501
BMI (kg/m^2^) ^‡^	23.0 ± 3.0	23.5 ± 2.9	0.762
Mean f/u (mon) *	24.6 (12–37)	24.6 (12–37)	0.791
Bilaterality, *n* (%) ^†^	18 (42.9)	9 (42.9)	-
Preop K-L grade, *n* (%) ^†^			
Grade III	11 (26.2)	5 (23.8)	0.838
Grade IV	31 (73.8)	16 (76.2)	
Current smoker, *n* (%) ^†^	3 (7.1)	8 (38.1)	0.002
Alcohol drinker, *n* (%) ^†^	3 (7.1)	10 (47.6)	<0.001
mCCI ^†^			0.022
2	-	-	
3 and 4	6 (9.5)	1 (4.8)	
5–8	28 (54.8)	9 (42.9)	
≥9	8 (35.7)	11(52.4)	
Hb level (g/dL) ^‡^	9.9 ± 1.3	11.2 ± 1.0	0.021
Total protein (g/dL) ^‡^	5.2 ± 1.2	5.9 ± 1.2	0.043
ASM index (ASM/height^2^), kg/m^2 ‡^	4.2 ± 0.5	5.8 ± 0.6	<0.001
Grip strength, kg ^‡^	15.4 ± 3.3	16.5 ± 3.1	0.021
6 m walking speed, m/s ^‡^	0.5 ± 0.2	0.9 ± 0.2	0.018

NOTE. BMI, body mass index; f/u, follow-up; Preop, preoperative; K-L, Kellgren–Lawrence; mCCI, modified Charlson Comorbidity Index; Hb, hemoglobin; ASMI, appendicular skeletal muscle mass index. * Values are provided as numbers with ranges. ^†^ Values are provided as numbers with percentages. ^‡^ Values are provided as means ± standard deviations.

**Table 4:** Sex differences of systemic and specific complications in propensity-matched cohort.

		Total (*n* = 63)	Female (*n* = 42)	Male (*n* = 21)	*p*-Value
	Cardiovascular	2 (3.2)	1 (2.4)	1 (2.4)	0.559
	Pulmonary	4 (6.3)	1 (2.4)	3 (14.3)	0.104
	Gastrointestinal	-	1 (2.3)	-	-
	Hepatic	6 (9.5)	4 (9.5)	2 (9.5)	0.686
Systemic	Nephrotic	-	-	-	-
	Endocrinologic	-	-	-	-
	Urologic	13 (20.6)	6 (14.3)	7 (33.3)	<0.001
	Cerebral	2 (3.2)	1 (2.4)	1 (4.8)	0.559
	Delirium	11 (17.5)	9 (21.4)	2 (9.5)	0.085
	Blood transfusion	17 (27.0)	12 (28.6)	5 (23.8)	0.466
	Venous thromboembolism				
	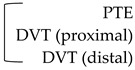	-	-	-	-
Specific		3 (4.8)	2 (4.8)	1 (4.8)	0.774
		6 (9.5)	4 (9.5)	2 (9.5)	0.686
	Infection	2 (3.2)	1 (2.4)	1 (4.8)	0.559
	Periprosthetic fracture	-	-	-	-

Note: PTE, pulmonary thromboembolism; DVT, deep vein thrombosis. Values are provided as number (percentage). DVT was classified as proximal or distal DVT. Thrombi limited to the popliteal vein or above were classified as proximal DVT, and thrombi within the calf vein were classified as distal DVT.

**Table 5:** Univariate and multivariate logistic regression analyses for the propensity-matched cohort.

Variable	*p* Value		Odds Radio (95% CI)	
	Univariate Analysis	Multivariate Analysis	Univariate Analysis	Multivariate Analysis
Age	0.501		1.8 (0.9–2.7)	
BMI	0.731		1.9 (1.8–2.2)	
Alcohol drinker	<0.001 *	0.003 ^†^	1.5 (0.8–1.7)	1.4 (0.9–1.8)
Current smoker	<0.001 *	0.019 ^†^	1.3 (1.1–2.5)	1.2 (0.9–1.4)
(Preoperative)				
K–L grade	0.751		1.2 (0.8–1.6)	
mCCI	0.012	0.036 ^†^	1.1 (0.8–1.4)	1.2 (0.8–1.5)
Hb level	0.021 *	0.253	0.8 (0.6–0.9)	0.7 (0.6–0.9)
Total protein	0.037 *	0.302	0.7 (0.5–0.8)	0.6 (0.5–0.8)
ASMI	<0.001 *	0.125	0.7 (0.5–0.8)	0.6 (0.5–0.8)

Note: BMI, body mass index; K-L grade, Kellgren–Lawrence grade; mCCI, modified Charlson Comorbidity Index; Hb, hemoglobin; ASMI, appendicular skeletal muscle index. * Univariate binary logistic regression analysis revealed that alcohol consumption, current smoker status, higher mCCI, preoperative Hb level, total protein, and ASMI were associated with the presence of male sarcopenia. ^†^ Multivariate binary logistic regression analysis showed that alcohol consumption, current smoker status, and higher mCCI were found to be predisposing risk factors for the presence of male sarcopenia.

## Data Availability

Data supporting the reported findings are available from the corresponding author upon reasonable request.
